# Paving the Way for Speech: Voice-Training-Induced Plasticity in Chronic Aphasia and Apraxia of Speech—Three Single Cases

**DOI:** 10.1155/2014/841982

**Published:** 2014-05-25

**Authors:** Monika Jungblut, Walter Huber, Christiane Mais, Ralph Schnitker

**Affiliations:** ^1^Interdisciplinary Institute for Music- and Speech-Therapy, Am Lipkamp 14, 47269 Duisburg, Germany; ^2^Clinical Cognition Research, University Hospital Aachen University, RWTH Aachen, Pauwelsstraße 30, 52074 Aachen, Germany; ^3^Aphasia Center North Rhine Westphalia, Laarmannstraße 21, 45359 Essen, Germany; ^4^Interdisciplinary Centre for Clinical Research—Neurofunctional Imaging Lab, University Hospital Aachen, Pauwelsstraße 30, 52074 Aachen, Germany

## Abstract

Difficulties with temporal coordination or sequencing of speech movements are frequently reported in aphasia patients with concomitant apraxia of speech (AOS). Our major objective was to investigate the effects of specific rhythmic-melodic voice training on brain activation of those patients. Three patients with severe chronic nonfluent aphasia and AOS were included in this study. Before and after therapy, patients underwent the same fMRI procedure as 30 healthy control subjects in our prestudy, which investigated the neural substrates of sung vowel changes in untrained rhythm sequences. A main finding was that post-minus pretreatment imaging data yielded significant perilesional activations in all patients for example, in the left superior temporal gyrus, whereas the reverse subtraction revealed either no significant activation or right hemisphere activation. Likewise, pre- and posttreatment assessments of patients' vocal rhythm production, language, and speech motor performance yielded significant improvements for all patients. Our results suggest that changes in brain activation due to the applied training might indicate specific processes of reorganization, for example, improved temporal sequencing of sublexical speech components. In this context, a training that focuses on rhythmic singing with differently demanding complexity levels as concerns motor and cognitive capabilities seems to support paving the way for speech.

## 1. Introduction

Functional imaging studies investigating therapy-induced recovery from aphasia after left-hemisphere stroke are rare (for review see [1]). This holds true even more for research with patients, who suffer from chronic nonfluent aphasia and concomitant apraxia of speech (AOS), a dysfunction of higher-order aspects of speech motor control characterized by deficits in programming or planning of articulatory gestures [2, 3].

Research results point out so far that neural correlates of functional recovery seem to involve both hemispheres. While in patients with small left-hemisphere lesions activation occurs to a greater extent in perilesional regions, in patients with large lesions involving the perisylvian language zone, there tends to be more activation of regions homologous to left-hemisphere language areas [4, 5]. Successful recovery seems to be correlated with perilesional activation; persistent right-hemisphere activation, however, seems to indicate slow and incomplete recovery [1, 6–11]. So far, only few studies demonstrated a direct impact of speech therapy on language recovery in chronic aphasia [12, 13].

The observation that even severely impaired aphasia patients are sometimes able to produce sung words more effectively than spoken words prompted many researchers and therapists to implement singing in the treatment of patients suffering from both motor speech disorders as well as aphasia [14–23]. Melodic intonation therapy (MIT), a form of speech therapy that was developed already in the 1970s, combines melodic intonation and rhythmic hand tapping with the objective to activate homologous language-capable regions in the right hemisphere [14, 22]. Neuroimaging research demonstrated conflicting results. The PET-study of Belin et al. included seven nonfluent aphasia patients, who received melodic intonation therapy (MIT) by comparing repetition of untrained MIT-loaded words to repetition of the same words spoken with a natural intonation [15]. Repetition of words using MIT strategies resulted in a significant increase of activation in left Broca's area as well as decrease in right-hemisphere regions. This result contradicts the essential objective of MIT to engage homologous right-hemisphere language regions. One criticism of this study is that activation changes were not measured by means of pre- and posttreatment image acquisition.

However, Schlaug et al. demonstrated treatment-associated fMRI changes in the right-hemisphere encompassing premotor, inferior frontal, and temporal lobes in a patient suffering from Broca's aphasia treated with MIT compared to a control patient [20]. Based on their post- and pretreatment results with diffusion tensor imaging, Schlaug et al. conclude that the right arcuate fasciculus can be remodeled by intense, long-term MIT [24].

As pointed out in detail in our previous studies, the greater bihemispheric organization for singing compared to speech might offer a chance for patients suffering from neurological speech and language disorders [25–27].

Singing combines pitch and intonation processing but also temporal processing and we are particularly interested in the latter. Temporal organization is an essential characteristic of language and speech processing and seems to be extremely vulnerable to left-hemisphere brain damage.

Lesion studies from the field of language as well as music demonstrate that patients with left-hemisphere lesions have problems with rhythm and time perception [28–31]. Many studies confirm deficits in aphasia patients with regard to temporal structuring of speech but also in AOS [3, 32, 69–71].

Stahl et al. investigated the importance of melody and rhythm for speech production in an experimental study with 17 nonfluent aphasia patients [33]. The authors conclude that rhythmic speech but not singing may support speech production. This applied particularly to patients with lesions including the basal ganglia.

Yet, timing deficits in these patient groups can be caused by very different reasons. While language production in aphasia patients may be nonfluent because of language-systematic reasons, for example, word retrieval deficits or agrammatic speech, temporal structuring in patients suffering from AOS is affected because of deficits in temporal coordination or sequencing of speech movements [34, 35]. Problems in accessing motor plans and programs result in temporal and prosodic distortions. Likewise, distortions of consonant and vowel segments are characteristic for AOS [36]. Extended segment and intersegment durations caused by disturbed anticipatory coarticulation result in slowed speech with visible and audible groping [37–39].

A number of treatment approaches from the field of speech therapy implemented strategies to control for rhythm or rate of speech production with patients suffering from AOS. Examples of such approaches are finger tapping [40], prolonged speaking [41], vibrotactile stimulation [42], metronomic pacing [3, 43, 44], or metrical pacing [45].

Taking the temporal organization of speech into account, we developed rhythmic-melodic voice training (SIPARI), a music therapy technique that is based on specific use of the voice [17, 25].

Over the past years, we performed several behavioral studies with patients suffering from chronic aphasia and AOS, which demonstrated that especially nonfluent patients significantly improved by this training [25, 26, 46, 47]. Since 2010, this treatment method is included in a Cochrane Review [48]. In a prestudy with 30 healthy control subjects, we investigated the neural substrates of chanted vowel changes in rhythm sequences by functional imaging in order to find explanations for the efficacy of this treatment [27]. Chanting is a rudimentary or simple form of singing, for example, on one pitch only.

According to our findings, rhythm structure is a decisive factor concerning lateralization as well as activation of specific, language-related areas during simple singing. With increasing demands on motor and cognitive capabilities additional activation of inferior frontal areas of the left hemisphere occurred, particularly in those areas, which are described in connection with temporal processing and sequencing [49–51]. These activations do not only comprise brain regions, whose lesions are causally connected with language disorders, but also regions of the left hemisphere (Broca's area, insular cortex, and inferior parietal cortex), whose lesions are reported to cause AOS [52, 53].

Our current study aims at investigating how the above-mentioned rhythmic-melodic voice training influences brain activation in patients with chronic nonfluent aphasia and concomitant AOS.

If it was possible to activate left-hemisphere language-related areas, as our imaging data with healthy subjects suggest, this might point to specific processes of reorganization, for example, improved temporal sequencing of sublexical speech components. Maybe, this explains at least in parts the efficacy of this treatment, which we already demonstrated in several behavioral studies mentioned above.

## 2. General Method

### 2.1. Patients

It is difficult to find relatively young and highly comparable patients. Therefore, only three patients with severe chronic nonfluent aphasia and concomitant AOS could be recruited from the Aphasia Center North Rhine Westphalia (Aphasiker-Zentrum NRW e.V.) for participation in this pilot study. Independently from the confirmation of the patients' therapists, three experienced speech therapists diagnosed the patients with AOS on the basis of direct observations involving inconsistently occurring phonemic and phonetic errors, initiation problems, prolonged segment durations, prolonged intersegment durations (sound/syllable/word segregation), disturbed prosody, visible groping, and effortful speech (see [36, 45]).

All patients were right-handed as determined by means of the Edinburgh Handedness Scale [54], German speaking, and were included in the study 18 months after the incident. None of the patients had premorbid history of neurological or psychiatric problems. They had no perceptual hearing impairments and their auditory comprehension was sufficient to understand the instructions. Their capacity regarding concentration and attention was good and their general health condition was stable enough for continuous participation during the 6-month treatment period of this research study.

Apart from general school education, none of the patients had any special musical training. All patients gave written consent to abstain from speech therapy in this study. All patients gave written informed consent in line with the Declaration of Helsinki and the Institutional Review Board of the RWTH Aachen. This study was undertaken in compliance with national legislation.

#### 2.1.1. Patient Mr. U

At the age of 53, Mr. U. suffered a left-hemisphere ischemic stroke involving in parts the area of the middle cerebral artery with secondary hemorrhage. The lesion encompassed frontotemporal regions. Clinical symptoms were spastic hemiparesis on the right and severe Broca's aphasia and moderate AOS. Up to the accident, Mr. U. was an industrial consultant in a leading position.

#### 2.1.2. Patient Mrs. A

At the age of 44, Mrs. A. suffered an extensive left-hemisphere cerebral hemorrhage encompassing frontotemporal regions and the area of caudate nucleus with extension to the basal ganglia as well as left internal capsule. Clinical symptoms were spastic hemiparesis on the right and severe global aphasia as well as severe AOS. Up to the accident, Mrs. A. was employed at an airline company.

#### 2.1.3. Patient Mr. H

At the age of 44, Mr. H. suffered an extensive left-hemisphere ischemic stroke in the area of the middle cerebral artery with displacement of the midline. The lesion encompassed frontotemporoparietal regions. Clinical symptoms were spastic hemiparesis on the right and both severe global aphasia and AOS. Up to the accident, Mr. H. worked as a bookseller and publisher.

A summary of the patients' characteristics is given in Table 1.

### 2.2. Stimuli and Procedure

Before and after therapy patients underwent the same fMRI procedure as 30 healthy control subjectsin our prestudy [27] in order to investigate if changes in brain activation occur due to the applied training.

**Figure 1:**
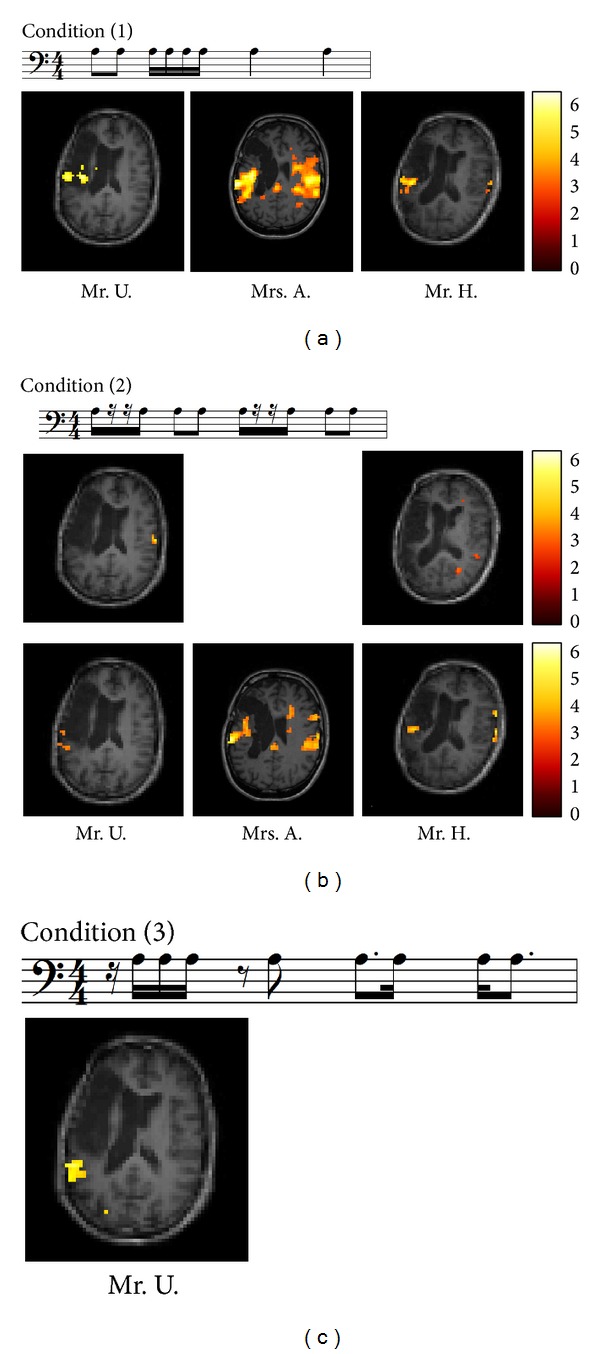
(a) Stimulus example of condition (1)* regular vowel changes* in musical notation. Quadruple measure groupings with a duration of 4 sec. (1 beat per sec.; 8 vowels alternately /a/i/) sung at a frequency of 220 Hz (A3), fMRI post-minus pretreatment results; (b) stimulus example of condition (2)* regular vowel changes* with rests in musical notation. Quadruple measure groupings with a duration of 4 sec. (1 beat per sec.; 8 vowels alternately /a/i/) sung at a frequency of 220 Hz (A3), fMRI results, upper row: pre-minus posttreatment and lower row: post-minus pretreatment; (c) stimulus example of condition (3)* irregular vowel changes* in musical notation. Quadruple measure groupings with a duration of 4 sec. (1 beat per sec.; 8 vowels alternately /a/i/) sung at a frequency of 220 Hz (A3) fMRI post-minus pretreatment results of Mr. U.

Tasks of our fMRI paradigm comprised repetition of chanted vowel changes in rhythm sequences with differently demanding complexity levels for the following reasons: chanting is a rudimentary or simple form of singing, for example, on one pitch only and facilitates evaluating the influenceof rhythm structure because melodic components are reduced. Rhythmic chanting (e.g., the vowel change /a/i/) requires exact temporal coordination and sequencing of speech movements. By focusing onsublexical processing with a single vowel change, we minimized the influence of semantic and lexical components of speech processing (for more details on the fMRI tasks see [27]). Stimuli consisted of quadruple measure groupings with duration of 4 sec. (8 vowels, alternately /a/i/) and differed as follows: (1) vowel changes with regular groupings, (2) vowel changes with regular groupings and rests, and (3) vowel changes with irregular groupings (see musical notations of Figures 1(a), 1(b), and 1(c)). Stimuli were sung by a female voice with the vowel change /a/i/ at a frequency of 220 Hz. Male patientswere encouraged to transpose the heard stimuli down an octave. The length of each stimulus was electronically set to 4 sec. with a max. deviation of 0.05 sec. For each condition (1–3) four different grouping variations were available.

Patients had to listen and to immediately repeat the heard stimuli after the presentation had stopped. We used an event-related design with a total of40 trials per condition and 40 randomly included null-events. The stimuli were presented in a pseudo-randomized order with a mean interstimulus interval of 9 sec. (jittered between 8 and 10 sec.). The presentation time took 4 sec. and the duration of the repetition period varied according to the estimated jitter time. The paradigm was implemented in Presentation (Neurobehavioral Systems) and synchronized to the scanner. Stimuli were presented binaurally through MR-compatible headphones with a sound absorption of 30 dBA (Resonance Technology). All conditions were performed with eyes closed.

Concerning movement artefacts, we point out that we compared three conditions utilizing the same response modality, that is, overt chanting. This allows generation of statistical maps that indicate activity more related to cognitive function than to movement [55]. Since our tasks are essentially demanding with regard to cognitive abilities (e.g., attention, short-term memory), which are often impaired in patients with frontal lobe damage, a sparse temporal scanning design was not used in this study. We wanted to avoid attention loss and consequently lower functional response caused by relatively long interscan intervals, which are required in sparse temporal schemes [56]. Moreover, stimuli were constantly sung at a frequency of 220 Hz, which is beyond the main frequency peaks of the scanner spectrum.

A remark in advance is that auditory stimulation was regarded as separately modeled condition in this design, which is not part of this paper. Auditory presentation and reproduction were time-shifted; patients did not sing along but after the presentation had stopped. Hence, the expected auditory activations in the auditory areas caused by the auditory stimulus presentation will not be present in the reported results (see [27]).

### 2.3. Musical Analyses

Recorded data of pre- and postrhythmic chanting of all patients were analyzed by 2 professional musicians (singer and percussionist) post hoc. They transcribed by ear and scored each stimulus repetition with either “correct” (score 1) or “incorrect” (score 0) regarding correct rhythm repetition. Tone repetitions (a total of 8 tones per stimulus) had to be timed correctly with a max. deviation of ±0.2 sec. each. Only unanimous assessments that rhythm production had been performed without error were scored 1. Comparison of pre- and posttreatment performance of the patients for each of the three tasks was statistically assessed by McNemar's test using exact binomial probability calculations (see Table 3).

### 2.4. Assessment of Language and Speech Motor Performance

Additionally, two experienced speech therapists of the Aphasia Center North Rhine Westphalia (Aphasiker-Zentrum NRW e.V.) who were blinded to the experiment performed two well-established diagnostic procedures for the German language as control tests at baseline and at the end of the 6-month treatment period in order to assess potential changes in language and speech motor capabilities.

#### 2.4.1. Aachener Aphasie Test

One instrument used for assessment of the efficacy of the treatment was the Aachener Aphasie Test (AAT) [57]. The AAT is a standardized procedure for evaluating type and severity of aphasia, developed and validated in the German language, subsequently translated into several European languages including English [58], and also validated and standardized in Dutch and Italian. The AAT may also be applied repeatedly in order to assess the efficacy of speech therapy interventions. The presence and type of aphasia were established using the ALLOC classification procedure, a nonparametric discriminant analysis computer program [57] using the normative data of the AAT. The AAT consists of six description levels for spontaneous speech (communicative verbal behavior, articulation and prosody, automatized language, semantic structure, phonemic structure, and syntactic structure) and five subtests (token test, repetition, written language, naming, and comprehension) for the assessment of specific language impairments. For an assessment of the degree of language impairment related to the entire group of aphasia patients, the AAT assesses percentile scores from the score values of the five subtests, that is, token test, repetition, written language, naming, and comprehension. The percentile score found for one test value indicates the percentage of patients of the exercise sample (*n* = 376) who have achieved the same or a lower score [57].

Although our primary focus was on expressive verbal behavior and motor speech performance, written language and comprehension were also assessed because reliable data regarding the speech profile can only be achieved if the AAT is administered in its complete version.

Apart from that, a more general transfer to other language modalities like written language and comprehension could not be excluded.

#### 2.4.2. Hierarchical Word List

Although not standardized, the hierarchical word list (HWL) is the first German diagnostic procedure, which allows systematic assessment of the symptoms caused by AOS [59]. The procedure contains a word/nonword repetition test (48 words and 48 matched nonwords) with word length varying between one and four syllables. Half of the items are phonologically simple (single consonants in syllable onsets or codas) and half are complex (consonant clusters in onsets or codas). All repeated words or nonwords (max. 96 items) are assessed in a quantitative analysis as regards* number* of assessable items,* phonetic structure, phonemic structure, *and* speech fluency.*

Qualitative analysis evaluates* speech effort, groping, syllabic speech, *and* deviant word accent* on a scale from 0 (without abnormality) to 3 (very pronounced) on an overall visual analogue scale. Each of these symptoms is precisely delineated in the HWL manual [59].

## 3. Data Analysis and General Procedure 

### 3.1. Image Acquisition

Functional images were obtained with a whole-body 3 T Siemens Trio MRI-system. Participants were fixated in the head coil using Velcro straps and foam paddings to stabilize head position and minimize motion artefacts. After orienting the axial slices in the anterior-posterior commissure (AC-PC) plane functional images were acquired using a T2*-weighted echo planar imaging (EPI) sequence with a repetition time (TR) of 2200 ms, an echo time (TE) of 30 ms, and a flip angle (FA) of 90 degrees. 640 volumes consisting of 41 contiguous transversal slices with a thickness of 3.4 mm were measured. A 64 × 64 matrix with a field of view (FOV) of 220 mm was used, yielding an effective voxel size of 3.44 × 3.44 × 3.74 mm.

### 3.2. Image Analysis

Functional images were preprocessed and analyzed using SPM8 (Wellcome Department of Cognitive Neurology London, UK). During preprocessing, images were realigned and unwarped in order to correct for motion and movement-related changes in magnetic susceptibility. Translation and rotation correction did not exceed 1.8 mm and 1.9°, respectively, for any of the participants. The anatomical T1 images of the patients were coregistered to the mean functional image using a rigid-body transformation implemented in SPM8 so that activation maps could be displayed on the structural images. As this study included patients with extended lesions, which may cause problems with the normalizing algorithm, images were not normalized into MNI space. Finally, all functional images were smoothed using a Gaussian filter of 8 × 8 × 8 mm to increase signal-to-noise ratio in the images [60].

### 3.3. Statistical Analysis

In the first-level statistical analyses, each preprocessed functional volume was entered into a subject specific, fixed-effect analysis using the general linear model approach for time-series data suggested by Friston and coworkers [60, 61] and implemented in SPM8. All stimulus onset times were modeled as single events.

Afterwards, stimulus functions were convolved with a canonical hemodynamic response function.

The data were high-pass filtered using a set of discrete cosine basis functions with a cut-off period of 128s in order to exclude low frequency confounds. For each of the 3 conditions of interest the contrasts of interest were generated. Statistical parametric maps (SPMs) were evaluated and voxels were considered significant if their corresponding linear contrast *t* values were significant at a voxelwise threshold of *P* = 0.05 (FDR-corrected). Only regions comprising at least 5 voxels will be reported.

### 3.4. General Procedure

For all patients therapy was started 18 months after the onset. None of the patients had ever received rhythmic-melodic voice training SIPARI before.

Each patient received 50 individual therapy sessions (60 minutes, twice a week) over a period of 25 weeks. During this period, no speech therapy took place. In order to control for comparable treatment conditions patients received exactly the same treatment program. We emphasize that none of the stimuli of the fMRI paradigm was trained during the treatment period of 6 months.

#### 3.4.1. Treatment Method

Therapy was conducted by the first author. The applied rhythmic-melodic voice training (SIPARI) comprises six components: singing, intonation, prosody, breathing (German: Atmung), rhythm, and improvisation. The efficacy of this treatment could be demonstrated in several behavioral studies with patients suffering from chronic aphasia and AOS [25, 26, 46, 47]. In 2010 a pseudorandomized controlled study with chronic nonfluent aphasia patients (mean duration of aphasia: 11,5 years), which examined the effects of the SIPARI method, was included in a Cochrane Review [48].

The main part of this treatment is based on specific use of the voice. Focusing initially on melodic speech components, which are mainly processed in the right hemisphere, a stepwise change to temporal-rhythmic speech components is carried out with the objective to stimulate phonological and segmental capabilities of the left hemisphere. To this end, an essential core of the treatment constitutes rhythmic singing with differently demanding complexity levels as concerns motor and cognitive capabilities. Since this treatment has been developed especially for severely impaired patients, an essential part of the verbal material comprises sublexical tasks (i.e., single vowels, vowel changes, consonant-vowel changes, etc.) in order to enable those patients to practice motor and cognitive function like planning, programming, and sequencing, that is, basics of language processing. The objective is a general transfer from the level of sublexical speech components to the level of words and phrases.

In terms of linguistics, SIPARI intervenes at the interface of phonological and phonetic encoding where access to the mental syllabary is supposed to take place [62, 63]. By embedding segmental and syllabic speech elements in rhythmic sequences with differently demanding complexity levels, specific grouping strategies are trained [64]. Apart from the fact that grouping or chunking (i.e., bundling events together into larger units) serves to enhance maintenance of information in working memory [65, 66], temporal-rhythmic chunking promotes speech motor processes by training intersyllabic programming, which is supposed to play an important role in phonetic planning [67]. In contrast to other treatment approaches mentioned in the introduction, which use pacing techniques or synchronous singing to an external timekeeper, SIPARI focuses on encouraging self-initiated planning and sequencing performance. Therefore, we give special emphasis on vocal training in connection with cognitive function, for example, executive control and working memory. This implies training of auditory short-term maintenance of melodic and rhythmic information in order to enable patients in a second step to coordinate the maintained information with verbal material.

Treatment objectives are to improve motor, linguistic, and cognitive functions and thus to support speech motor processes and also language-systematic processes, that is, to encourage planning, programming, and sequencing.

An appendix containing the treatment interventions can be provided. We did not include a description of the method because detailed information on the method as well as selection of exercises has been already published elsewhere [17, 25, 46, 68].

## 4. Results

### 4.1. fMRI Data

To determine how neural activity differed before and after therapy, both subtractions (i.e., pre-minus post-therapy and post-minus pre-therapy) were performed for all conditions. The anatomical localizations were determined by two experienced experts (neuroanatomist and neuroradiologist) from the University Hospital Aachen.

#### 4.1.1. Subtractions for Condition (1) Vowel Changes with Regular Groupings

Subtraction pre-minuspost-therapy yielded no significant activation for any patient.

(1)* Mr. U.* Subtraction post-minus pre-therapy yielded significant activation in the left hemisphere, comprising the basal ganglia (caudate nucleus), insula, and inferior frontal regions. Left superior temporal gyrus was also activated significantly.

Comparison with the anatomical image showed that Mr. U. has a tissue bridge in the infarcted area.

(2)* Mrs. A. *Subtraction post-minus pre-therapy demonstrated significant activations in both hemispheres, including superior and middle temporal gyrus. Further activation was also found in the left insula. Precentral gyrus was activated in the right hemisphere.

(3)* Mr. H. *Subtraction post-minus pre-therapy yielded bilateral activation of the superior temporal gyrus, however, more pronounced in the left hemisphere.

#### 4.1.2. Subtractions for Condition (2) Vowel Changes with Regular Groupings and Rests

(1)* Mr. U. *While subtraction pre-minuspost-therapy demonstrated significant activation in the right precentral gyrus, the reverse subtraction yielded a shift of significant activation to the left precentral gyrus and superior temporal gyrus.

(2)* Mrs. A. *Subtraction pre-minuspost-therapy yielded no significant activation. The reverse subtraction, however, showed significant activation of the left superior temporal gyrus and bilateral inferior frontal gyrus. Middle temporal gyrus and cingulate gyrus were activated in the right hemisphere.

(3)* Mr. H. *Pre-minuspost-therapy subtraction showed activation in the right middle and superior temporal gyrus. The reverse subtraction demonstrated significant bilateral activation in the superior temporal gyrus and precentral gyrus activation in the right hemisphere.

#### 4.1.3. Subtractions for Condition (3) Vowel Changes with Irregular Groupings

For Mrs. A and Mr. H., neither subtraction pre-minuspost-therapy nor the reverse subtraction showed any results.

*Mr. U. *While no significant activation could be found in subtraction pre-minuspost-therapy, the reverse subtraction yielded significant activation in the left hemisphere comprising the superior and middle temporal gyrus.

With regard to anatomical locations, cluster sizes, and *t* values see Table 2.

### 4.2. Behavioral Analysis

#### 4.2.1. Musical Analyses

Recorded data of pre- and post- rhythmic chanting of all patients were analyzed by 2 professional musicians (singer and percussionist) post hoc. Only unanimous assessments that rhythm production had been performed without error were scored 1. Overall interrater agreement resulted in 6 = 0.99, *P* < 0.002. Comparison of pre- and posttreatment performance of the patients for each of the three tasks was statistically assessed by McNemar's test using exact binomial probability calculations (see Table 3). All patients improved statistically significant (*P* < 0.001) in condition (1)* vowel changes with regular groupings. *Analyses of condition (2)* vowel changes with regular groupings and rests* yielded statistically significant improvements for Mr. U. (*P* < 0.01) and Mr. H. (*P* = 0.01). Mr. U. achieved a statistically significant improvement (*P* < 0.012) in condition (3)* vowel changes with irregular groupings.*

#### 4.2.2. Aachener Aphasie Test (AAT)

Clinically significant improvements could be assessed in the subtests token test, repetition, and naming. Further significant improvements were achieved regarding changes in profile level and spontaneous speech.

#### 4.2.3. Hierarchical Word List (HWL)

Quantitative analysis revealed improvements concerning* number* of assessable items (Mr. H.),* phonetic structure *(Mr. U. and Mr. H.),* phonemic structure *(Mr. U. and Mr. H.), and speech* fluency* (Mrs. A. and Mr. H.).

Qualitative analysis yielded for Mr. U. less speech effort and less syllabic speech (improvement of 1.5 points each out of an overall scale of 3 points) and for Mrs. A. and Mr. H. less speech effort and less groping (improvement of 1 point each).

## 5. Discussion 

Difficulties with temporal coordination or sequencing of speech movements are frequently reported in patients suffering from aphasia and AOS [3, 32, 69–71]. Our own experiences are in accordance with these findings and prompted us to develop rhythmic-melodic voice training SIPARI [17, 25], which was applied in this study (see Sections 1 and 3.4.1). 

The major objective of this pilot study was to investigate how this training influences brain activation in three patients with severe chronic nonfluent aphasia and AOS (1 Broca's, 2 global aphasia patients). Before and after therapy each patient underwent the same fMRI procedure as 30 control subjects in our prestudy [27].

To determine how neural activity differed before and after therapy, both subtractions (i.e., pre-minus post-therapy and post-minus pre-therapy) were performed for all three conditions (see Section 4.1 and Figures 1(a), 1(b), and 1(c)). In addition, pre- and posttreatment results of patients' vocal production as well as their language and speech motor performance were examined by cognitive methods.

### 5.1. Rhythm Production and Brain Activation

All patients improved most in* condition (1) vowel changes with regular groupings.*

Musical analyses of the recorded data revealed that before therapy none of the patients had any strategy to manage the demands of this condition. It should be mentioned that this condition comprises either no vowel change within one beat or the same tone durations and regular changes within one beat. From beat to beat tone durations change in even-numbered ratios (see musical notation of condition (1) Figure 1(a)). A conceivable strategy to keep the respective stimulus in short-term memory in order to reproduce it afterwards could be, for example, to group vowel changes on the basis of tones with equal durations [64, 72, 73]. We suggest that due to therapy patients could use an adequate strategy in the post-therapy assessments more effectively. First, musical analyses corroborate this assumption. Secondly, subtraction post-minus pre-therapy resulted in brain activation comprising areas, which are described not only in connection with temporal processing and sequencing [49–51] but also with language and speech processing, for example, inferior frontal gyrus, insula, basal ganglia (caudate nucleus), and particularly superior temporal gyrus [1, 74–76].

However, while significant activations of the Broca's aphasia patient (Mr. U.) were found exclusively in the left hemisphere, in both global aphasia patients (Mrs. A. and Mr. H.) significant activations were measured in perilesional and also in homologous areas in the right hemisphere (see Figure 1(a)). Moreover, comparison of all three conditions points to increases or changes of activation that differ depending on task demand; for example, for all patients, activation was most pronounced in this post-minus pre-therapy comparison. Task-dependent activation changes are in line with our prestudy with healthy subjects [27]. However, these findings are all the more remarkable as our study also included chronic global aphasia patients with large lesions. Since Mr. H. also improved in basically all measures regarding his language performance a possible explanation could be that in contrast to the other two patients at least parts of his arcuate fasciculus are still intact. However, we cannot verify this assumption because diffusion tensor imaging data are not available.

Further research is needed, especially if we compare our results with other studies, which investigated the therapeutic effect of singing on language rehabilitation. For instance, Schlaug et al. demonstrated treatment-associated fMRI changes in the right-hemisphere encompassing premotor, inferior frontal, and temporal lobes in a patient suffering from Broca's aphasia treated with melodic intonation therapy (MIT) [20]. Based on their post- and pretreatment results with diffusion tensor imaging, Schlaug et al. even conclude that the right arcuate fasciculus can be remodeled by intense, long-term MIT [24].

Musical analyses of* condition (2) vowel changes with regular groupings and rests* demonstrated that Mr. U. and Mr. H coped better with this task already at the beginning of the treatment (see Table 3). Comparison of image analyses revealed for Mr. U. a shift of activation from right precentral gyrus in the pre-minus-posttreatment subtraction to left precentral gyrus and superior temporal gyrus activation in the reverse subtraction (see Figure 1(b)). The same way, data of Mr. H. demonstrate that activation changed from right middle and superior temporal gyrus activation in the pre-minus-posttreatment subtraction to bilateral superior temporal gyrus activation in the reverse subtraction. Additional activation could be measured in the right precentral gyrus. What is special about this condition is that implementation of rests brings about higher demands on timing because legato and staccato vocalization changes from beat to beat (see musical notation of condition (2) Figure 1(b)). This way of vocalization requires precise execution of articulatory movements because staccato and legato vocalizations change from beat to beat. Particularly, the initial phase of vocal preparation becomes the focus of attention, which is reported to be dominated by the left hemisphere [77]. Since findings of our prestudy with healthy subjects also corroborate this assumption [27], this may explain the shift from right to left superior temporal and precentral gyrus (Mr. U.) representing improved auditory-motor interaction in a task, which requires exact executed vocalization. This may also hold true for Mr. H., who additionally activated left superior temporal gyrus but had to compensate with regard to motor preparation by activating right precentral gyrus due to his lesion in the left homologue. Musical evaluations confirmed more correct entries and improved legato and staccato differentiation in the post-therapy analyses. While Mrs. A. was not able to manage this task before therapy, musical evaluation as well as post-minus pre-therapy subtraction point to improved planning with significant activations in the left superior temporal gyrus. Bilateral inferior frontal gyrus activation but also activation of right cingulate gyrus suggest that this task was demanding for Mrs. A. Based on the results of our prestudy [27], we assume that these activations are related not only to sustaining attention in order to maintain temporal information in memory [78] but also to coordination of response generation and respective action planning [79].

The only patient, who developed a strategy to manage c*ondition (3) vowel changes with irregular groupings* post-therapy, was Mr. U. His post-minus pre-therapy subtraction data yielded significant left-hemisphere activation in the superior and middle temporal gyrus (see Figure 1(c)). Since irregularity of this condition caused by implementation of syncopations, dottings, and rests further increases the demands on auditory-motor interaction, activity seems to be focused on this area, which is reported to be interfacing with motor planning systems for sublexical aspects of speech [75, 76]. One may object that posterior superior temporal gyrus activation in basically all of our post-minus pre-contrasts, with an asymmetry towards the left, might only indirectly reflect any improvements but merely auditory processing. This may hold true if we had limited our analysis to the fMRI data only. However, language and speech motor outcomes were additionally tested by blinded assessors.

### 5.2. Improvements of Language and Speech Motor Capabilities

**Figure 2:**
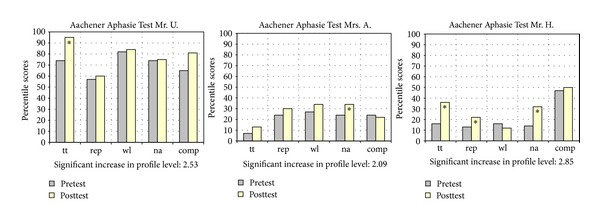
Results of the Aachener Aphasie Test (AAT) (in percentile scores) and changes in profile level (in t-scores); ∗ indicates significant improvement tt = token test, rep = repetition, wl = written language, na = naming, and comp = comprehension.

Post- and pretest comparisons revealed clinically significant improvements for all patients in the Aachener Aphasie Test (AAT) concerning the subtests Token Test (Mr. U. and Mr. H.), repetition (Mr. H.), and naming (Mrs. A. and Mr. H.). Furthermore, all patients achieved a clinically significant increase in profile, thus testifying to the fact that an improvement in the overall range of all five subtests occurred (see Figure 2). These improvements are remarkable as they concern expressive language capabilities (in particular naming) of two severely impaired global aphasia patients (Mrs. A., Mr. H.). Particularly in connection with further improvements in the Token Test (Mr. U., Mr. H.), which represents a measure to evaluate the severity of the aphasic disorder, these results imply that more comprehensive activation of language-systematic processes must have been initiated. Likewise, this assumption is corroborated by substantial improvements in spontaneous speech for all patients (see Table 4). Our findings are in line with our previous therapy studies [25, 26, 46, 47]. We suggest that specifically focusing on improving cognitive function (e.g., auditory short-term and working memory performance), which is one of the main objectives of the applied treatment, is an essential reason for these improvements (see Section 3.4.1 and [17]).

**Figure 3:**
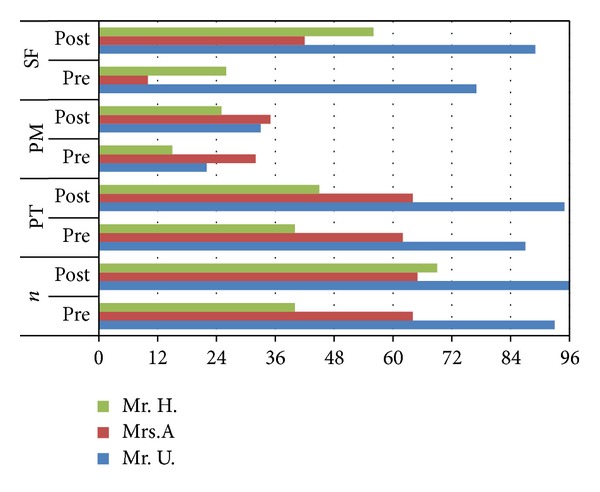
Quantitative analysis of the hierarchical word list (HWL), *n* = number of assessable items (max. 96 items); PT = phonetic structure; PM = phonemic structure; and SF = speech fluency.

Moreover, assessments of speech motor capabilities of the patients revealed improvements concerning* number* of assessable items (Mr. H.),* phonetic structure *(Mr. U. and Mr. H.),* phonemic structure *(Mr. U. and Mr. H.), and speech* fluency* (Mrs. A. and Mr. H.) (see Figure 3).

All patients produced the items with significantly less speech effort in the posttest, two of them, namely, the global aphasia patients with severe AOS (Mrs. A. and Mr. H.), also with less groping.

The improvements concerning* phonetic structure* and* phonemic structure* are remarkable insofar as they indicate that not only retrieval of motor plans for phones but also sequential organization of movements for a series of phones improved, exactly those processes that are particularly impaired in patients with AOS [34, 80]. Likewise, one frequently cited temporal characteristic of apraxic speech is a reduction in overall speech rate [34]. Since qualitative analysis yielded that all patients improved in* speech fluency,* it would appear that also patients with AOS benefit from a treatment, which combines motor and cognitive training.

## 6. Conclusion

In this therapy study including three patients with chronic nonfluent aphasia and AOS, we demonstrated the effects of rhythmic-melodic voice training (SIPARI) by functional imaging. While post-minus pretreatment imaging data of the Broca's aphasia patient (Mr. U.) yielded significant left-hemisphere activation in perilesional regions, activation patterns of both global aphasia patients (Mrs. A. and Mr. H.) comprised perilesional regions as well as homologous areas in the right hemisphere. A neural correlate of a system, which is supposed to interface with motor planning systems for sublexical aspects of speech [75, 76], was consistently located in the left superior temporal gyrus. This auditory-motor circuit provides the essential neural mechanisms for phonological short-term memory [81, 82]. Functional reintegration of this region is mentioned in the literature in connection with language improvement [1, 10, 83–85].

Although patients of our study are already in the chronic stage and have large lesions, they improved significantly with regard to language but also speech motor capabilities. They recruited parts of the neural network that we previously found in healthy subjects using the same fMRI paradigm, for example, inferior frontal gyrus, insula, and basal ganglia [27]. In addition, our findings indicate that also in severely impaired patients activations vary with task demand. These results are new and significant in particular for directed therapy interventions. Therefore, further research will elucidate potential influences in greater detail, for example, the relationship between rhythm structure, grouping strategy, and phonological working memory.

Based on our results, we assume that, for example, an improvement of short-term storage of sublexical phonological material and, as a result of this, improved temporal sequencing possibly represent one essential prerequisite for improvements of speech motor but also language capabilities. Planning, programming, and sequencing include motor as well as cognitive capabilities. In this context, the singing voice may serve as a gateway be it that linguistic as well as musical components are applied systematically.

## Figures and Tables

**Table 1:** Summary of the patients' characteristics. CCTs (obtained within 3 days after the cerebral accident); the left side of the brain is on the right.

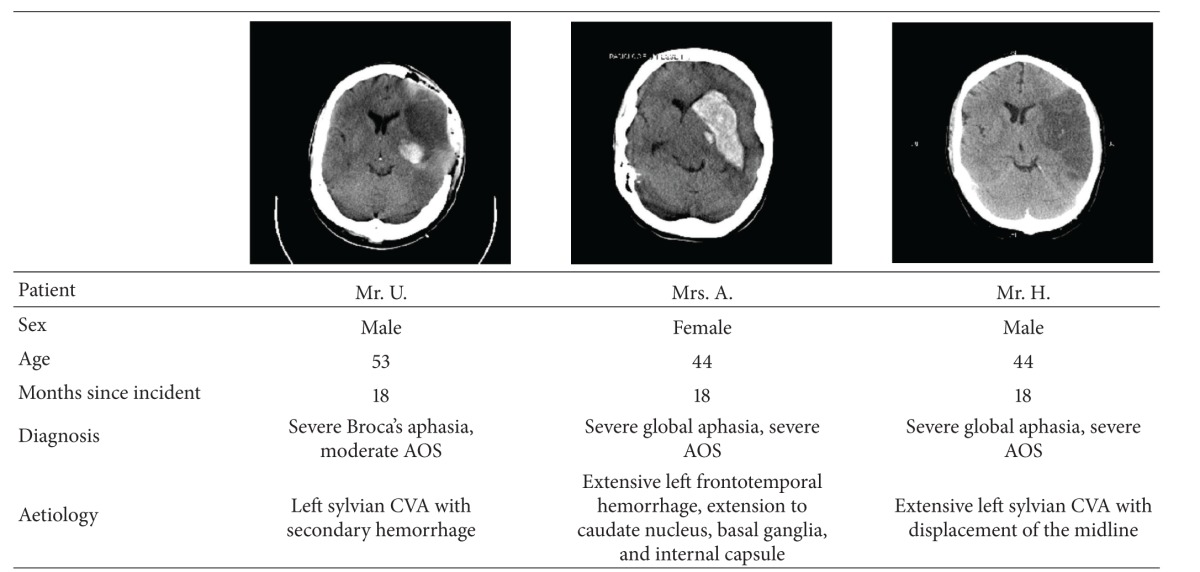

AOS: apraxia of speech; CVA: cerebral vascular accident.

**Table 2:** Anatomical location, cluster sizes (*k*, number of voxels), and *t* values of areas of significant activation.

	Condition 1				Condition 2				Condition 3			
	Anatomical location	Side	Cluster size *k*	*t* value	Anatomical location	Side	Cluster size *k*	*t* value	Anatomical location	Side	Cluster size *k*	*t* value
Mr. U	Subtraction post-minus pre-therapy				Subtraction pre-minus post-therapy							
	Insula	L	119	4.64	Precentral gyrus	R	19	4.49				
	Inferior frontal gyrus	L	28	4.01								
	Superior temporal gyrus	L	20	3.92								
	Nucleus caudatus	L	99	4.89								
					Subtraction post-minus pre-therapy				Subtraction post-minus pre-therapy			
					Precentral gyrus	L	9	3.69	Middle temporal gyrus	L	71	5.34
					Superior temporal gyrus	L	11	3.62	Superior temporal gyrus	L	24	4.07

Mrs. A	Subtraction post-minus pre-therapy				Subtraction post-minus pre-therapy							
	Superior temporal gyrus	R	2737	5.4	Superior temporal gyrus	L	143	4.89				
	Middle temporal gyrus	R			Inferior frontal gyrus	L						
	Superior temporal gyrus	L	341	5.16	Middle temporal gyrus	R	11	3.34				
	Middle temporal gyrus	L			Inferior frontal gyrus	R	14	3.31				
	Insula	L			Cingulate gyrus	R	31	3.78				
	Precentral gyrus	R	15	3.56								

Mr. H	Subtraction post-minus pre-therapy				Subtraction pre-minus post-therapy							
	Superior temporal gyrus	L	42	5.37	Superior temporal gyrus	R	16	3.59				
	Superior temporal gyrus	R	7	4.81	Middle temporal gyrus	R						
					Subtraction post-minus pre-therapy							
					Superior temporal gyrus	L	12	4.48				
					Superior temporal gyrus	R	28	5.07				
					Precentral gyrus	R	15	4.60				

**Table 3:** McNemar's test comparison of pre- and posttreatment rhythmic chanting (40 tasks per each condition) condition (1) regular vowel changes; condition (2) regular vowel changes with rests; condition (3) irregular vowel changes.

	Mr. U		Mrs. A		Mr. H	
Condition 1	14	22	16	20	17	20
	0	4	1	3	2	1
*P*=	Two-tailed	**<0.001**	Two-tailed	**<0.001**	Two-tailed	**<0.001**

Condition 2	7	17	28	8	10	18
	4	12	1	3	5	7
*P*=	Two-tailed	**<0.01**	Two-tailed	Not sign.	Two-tailed	0.01

Condition 3	27	10	37	0	33	4
	1	2	1	2	3	0
*P*=	Two-tailed	<0.012	Two-tailed	Not sign.	Two-tailed	Not sign.

	No—no	No—yes				
	Yes—no	Yes—yes				

**Table 4:** Spontaneous speech.

		cvb	ap	al	sem s	ps	syn s
Mr. U.	Pre	2	2	3	3	2	2
	Post	3*	3*	4*	4*	3*	2

Mrs. A.	Pre	1	1	2	2	2	1
	Post	2*	2*	2	2	2	1

Mr. H.	Pre	1	1	1	0	0	0
	Post	2*	2*	2*	1*	2**	1

*substantial; **significant.

Aachener Aphasie Test, evaluation of spontaneous speech; min = 0, max = 5 points.

cvb: communicative verbal behavior; ap: articulation and prosody; al: automatized language.

sem s: semantic structure; ps: phonemic structure; syn s: syntactic structure.
